# Impact of special economic zones on socioeconomics and local development in Pakistan: Evidence from Allama Iqbal Special Economic Zone, Faisalabad

**DOI:** 10.1371/journal.pone.0310488

**Published:** 2024-11-14

**Authors:** Shahid Karim, Kong Xiang, Abdul Hameed

**Affiliations:** 1 School of Geographical Sciences, East China Normal University, Shanghai, China; 2 Department of Geography, Government College University, Lahore, Pakistan; 3 The Centre for Modern Chinese City Studies, East China Normal University, Shanghai, China; 4 Federal Urdu University of Arts, Science and Technology, Islamabad, Pakistan; Balochistan University of Information Technology Engineering and Management Sciences, PAKISTAN

## Abstract

The purpose of this study was to investigate the impact of SEZ on indigenous peoples’ socioeconomic status and local development in the study area. A quantitative approach to analyzing the socioeconomics of treatment and control groups. A structured questionnaire was designed and a field survey was undertaken to collect primary data from respondents. This study used Principal Component Analysis (PCA) to create a socioeconomic index for two groups: those who sold their agricultural land and those who did not sell, and a two-sample independent t-test was used to determine the influence of SEZ on socioeconomic and local development. The results showed that the compensation amount for the acquired land not only improved the socio-economic living conditions of the indigenous population in short run, but also transformed their type of employment from agriculture to labor work, increased health expenditure, increased household wealth and minor changes in education expenditure and construction effected new houses, most of which is used for child marriage, vehicle purchased and dowary expenses in the special economic zone. This unproductive spending increases in the short term, which in the long run will convert skeikonicity into deprivation. Previous studies focused only on the geopolitics behind the geo-economy and the challenges and success factors for SEZs in Pakistan. This study is unique as it is the first attempt that uses statistical and economic tools to identify the positive and negative impacts of SEZ on the local development in the area. It makes an academic contribution to the literature to improve the knowledge of the effects of these special economic zones on the local development in any area.

## 1. Introduction

Presently, around 700 million people worldwide live in extreme poverty. Over time, there has been a decreasing trend in the number of poor people globally. This decline is mainly due to the rapid economic growth in countries with large populations like China and India [1]. However, there are significant differences among countries and regions in the developing world. Certain regions and countries in places like East Asia are rapidly progressing towards industrialization, similar to developed nations. On the other hand, some areas, particularly in Sub-Saharan Africa, are significantly behind, and in certain countries, the proportion of poor people has even increased. The growth of industries has played a crucial role in the economic advancement of nations. While some countries have achieved economic growth while ensuring fairness, in others, inequality has remained high [2].

Several countries have tackled these challenges by implementing an industrial strategy by the creation of special economic development zones (SEZs) [3]. SEZ is a phrase that refers to different types of business zones [4]. SEZs are defined by a demarcated geographic region, local administration, specific benefits, unique traditions and administrative procedures. Working under more liberal laws and regulations than in other parts of the country offers its own set of benefits [5].

The SEZs have had a significant economic impact in the developing world, especially in the People’s Republic of China. Within the Chinese economy, the SEZs have yielded significant benefits. They have aided in the decentralization of resources and the establishment of an open market economy. They aided in the attraction of Foreign Direct Investment (FDI) and international enterprises, both of which have strengthened the economy and contributed to raising the standard of living [6, 7]. One of the most significant economic effects of SEZs on the local economy has been the level of income with an increased salary and earning potential of workers inside the region [8, 9]. After Chinese success, many countries such as Sri Lanka, India, Bangladesh and Cambodia adopted this policy to achieve the key benefits of SEZs. e.g. a major tool of job creation in Sri Lanka [10] tax rebates, fiscal incentives, and land at subsidized rates in India [11] large-scale investments, a massive job creation (32% per year) and an increased (81%) FDI inflow in Bangladesh [12] and a reduced income disparity especially for the female employees at the district level in Cambodia [13, 14].

Keeping in view the success of these SEZs in developing nations of Asia, especially in China and Bangladesh, the government of Pakistan has decided to establish nine economic zones under the umbrella of the China–Pakistan Economic Corridor (CPEC), which is the flagship project of the Belt and Road Initiative (BRI). Most of the recent literature on SEZs [15–36] concentrated on the impact of SEZs on foreign direct investment (FDI), trade policy, challenges and success factors for SEZs, firm productivity, exporting behavior, SEZs boosting investment attractiveness and institutional improvement. All of these policy sectors need research to play a significant role in national and sub-national economic, social, and political growth. The fundamental aims of these SEZ initiatives worldwide were to encourage local development and eradicate chronic poverty, food insecurity, and economic inequality as well as country economic growth [8–14]. The prior development of SEZs in developing countries often goes unnoticed. Before the infusion of economic growth, SEZs encountered numerous local-level challenges such as selecting suitable areas, establishing local infrastructure, acquiring land, training local labor, and addressing legislative concerns. These challenges significantly contributed to the successes of SEZs.

Furthermore, in some scenarios, certain SEZs have been planned or developed on productive agricultural land that was previously utilized by farmers for cultivating cash crops or staple food grains. For instance, in Pakistan, the Allama Iqbal Special Economic Zone in Faisalabad was planned in an area predominantly occupied by farmers whose land was acquired for this SEZ. Due to the weak policies and strategies of the Government of Pakistan, agricultural land was obtained from the farmers, resulting in the current state of the SEZ being pending and non-operational. Conversely, researchers speculate that the farmers received monetary compensation but utilized it for personal consumption without yielding any economic benefits. Consequently, the Pakistani government expended a substantial amount of money without achieving any gains, leaving the SEZ non-functional.

The preceding investigations, to some extent, ignored this component of SEZs. Some studies, particularly in Pakistan have been conducted by [3, 14, 19, 22, 23, 25, 29–31, 36] to study these issues regarding SEZs and CPEC. These studies primarily concentrated on geopolitics and geo-economics as well as the challenges and success factors for SEZs, while largely ignoring local development in SEZ regions. In this sense, our work is unique in the way that it bridges the gap between the socioeconomic implications of SEZs and the local development in Pakistan. Furthermore, being empirical in nature, this is the first study to employ the updated primary survey data to capture the true impact of SEZs on the socioeconomic and local development of the local populations before its operationalization.

The primary objectives of this study are as follows:

- Evaluate the influence of monetary compensation received by farmers on their well-being.
- Determine the effect of SEZs’ land acquisition on the local development of indigenous farmers
- Estimate the socioeconomic impact of SEZs on the farming community.

The importance of this study stems from its reaction to the changing environment of SEZs in developing countries, notably within the context of the CPEC as part of the BRI Initiative. Poverty reduction is a primary goal of equitable and sustainable development, which has received extensive attention from the academia [37]. There has been a significant vacuum in studying the influence of SEZs on local development and socioeconomics. This study aims to bridge this gap by delving into the overlooked facets of SEZ development, particularly in Pakistan. By evaluating the impact of monetary compensation on farmer well-being, examining the influence of SEZ land acquisition on local development, and estimating the socioeconomic effects on the farming community, this research aims to provide a comprehensive understanding of the direct consequences of SEZ implementation. Moreover, this study is distinguished by its empirical approach, utilizing updated primary survey data to capture the genuine impact of SEZs on local communities before their operationalization. By filling this research void, the study seeks to contribute valuable insights that could inform policymakers, guiding more inclusive and beneficial strategies for SEZ development in Pakistan.

The remainder of this manuscript’s contents is organized as follows. Section 2 describes the Literature review, section 3 deals with materials and method, section 4 presents the results of empirical estimation-related elements, section 5 discusses the key elements that are emerging and are being discussed and section 6 concludes the paper by summarizing the conclusions and policy implications.

## 2. Literature review

The function of SEZs and local development programs in determining socioeconomic growth and alleviating regional inequities is critically examined in this research literature review. SEZs, which are recognized as economic reform and development instruments, have attracted global attention for their ability to attract investments, support industrial expansion, and accelerate economic advancement in certain geographical locations. Myrdal (1957), stated that regional inequalities occur from unequal access to resources, resulting in underdevelopment in certain regions despite overall economic growth. Gunnar Myrdal’s regional differences hypothesis ascribed this disparity to market forces and geographic advantages driving economic growth in certain locations [38]. Shankar (2003), shows that less developed countries, on the other hand, frequently suffer owing to intrinsic limits such as a lack of human capital, technical improvements, and fiscal resources, necessitating interventions outside market mechanisms [39].

According to Fan, Kanbur, & Zhang (2011), infrastructure development, social protection programs, and governance reforms are examples of policy initiatives targeted at eliminating regional disparities [40]. Zia & Waqar (2018), concluded that SEZs have emerged as a strategic instrument for addressing these imbalances by attracting investments and stimulating economic activity [3]. Crane, Albrecht, Duffin, & Albrecht (2018), stated that to attract firms, SEZs, which are defined by different geographical zones with distinct legislation and economic policies, provide incentives such as tax breaks, infrastructure development, and streamlined administrative processes [5].

Sharma (2009) and Wang (2013), resulted that SEZs have been shown in studies throughout the world to have a favourable influence on local economies. SEZs have greatly strengthened the Chinese economy by attracting foreign investment, enhancing living standards, and expanding job possibilities [7, 9]. Similarly, Abeywardene et al., (1994), Farole & Akinci (2011), Kulkarni & Sanyal (2016), concluded that SEZs have contributed significantly to job creation and economic growth in Sri Lanka, Bangladesh, and India [10–12]. However, Matias et al., (2013), empirical research suggests that the efficiency of SEZs varies. While some studies show beneficial benefits on employment and incomes with no negative effects on housing or population [16]. Alkon (2018), mentioned other obstacles, such as land conflicts and insufficient local development spillovers [17].

Rao and Ibáez (2005), presented on a detailed research of Jamaica, their mission investigated the impact of the Jamaica Social Investment Fund. They examined the lives of 500 household and over 700 individuals using an inventive combination of quantitative and qualitative tools. They discovered remarkable insights through semi-structured conversations with local leaders and thorough data analysis. Their findings reflected stories of growing trust, collaboration across various populations, and poverty-reduction tendencies. Nonetheless, socioeconomic gaps cast a shadow over these achievements, distorting the quantitative consequences [41].

Matias et al., (2013), set out to explore the impact of the federal urban Empowerment Zone (EZ) program. Their expedition unearthed a treasure trove of revelations. They revealed the program’s ability to generate jobs, elevate local earnings, all without triggering significant population or housing rent increases. Their insights added to the lore surrounding the effectiveness of place-based policies in fostering job creation [16].

Wang (2013) set out to study how Special Economic Zones (SEZs) affected China’s local economy. By carefully comparing cities created at different times, Wang found that SEZs led to more foreign investment, stronger local economies, and higher salaries. However, he also noticed that zones established later had fewer benefits and caused more problems in business behavior [9].

Kulkarni and Sanyal (2016), constructed a tome of knowledge as the stories unfolded, highlighting India’s history with SEZs. Their story took them through policies that promote industrial expansion, as well as the relationship between SEZs, sustainable development, and local geographical variations. They emphasized the need of infrastructure for long-term growth [11]. Fan, et.al. (2011), explored China’s socioeconomic landscapes. They suggested using more experimental methods, praised the success of regional development programs, but also warned about the growing influence of market forces [40].

Alkon’s (2018), description of India’s SEZs, on the other hand, throws doubt on their usefulness, demonstrating unfulfilled promises in local socioeconomic growth [17]. Glaeser & Gottlieb (2008), analytical study digs into the complex interplay between labour mobility and the efficacy of place-based policies such as SEZs, including the possible influence of perfect labour mobility on the welfare benefits received from such policies [42].

While global research recognises SEZs as critical tools for attracting investments, driving economic growth, and reducing regional disparities, numerous empirical studies, such as those conducted in China, Jamaica, the United States, and India, present varying results regarding SEZ effectiveness. While some studies demonstrate beneficial effects on job creation, income growth, and economic advancement, others show obstacles such as land conflicts, limited local development spillovers, and unmet social growth claims.

The variations in findings highlight the difficulties and variability of SEZ outcomes in Pakistan, indicating a need for a more understanding of the factors determining their success or failure. A gap in the literature exists regarding the assessment of local development prior to SEZ implementation. New research focused on this aspect will greatly contribute to closing these gaps by providing insights into crucial elements such as infrastructural preparedness, policy efficacy, and potential regional barriers. Such a study is essential for developing informed policies that maximize the benefits of SEZs while addressing the unique socioeconomic dynamics of Pakistan’s regions, promoting equitable and sustainable development.

## 3. Materials and methods

### 3.1. Study area

District Faisalabad spans an area of 5857 square kilometers and is home to a total population of 7.9 million individuals, with 4.11 million residing in rural areas and 3.76 million in urban areas. The population density, based on the 2017 census of Pakistan, is 1345 persons per square kilometer. The district, situated between the Ravi and Chenab rivers, is a significant agricultural area in Pakistan, known for its cultivation of wheat, sugarcane, and rice, which are the primary cereal and cash crops [43]. The District is renowned in Pakistan for its thriving textile, hosiery, and apparel industry. The notable sectors are Rice and Oil Mills, Chemical Industries, Tiles and Ceramics, Electronics, and Car Manufacturing. Moreover, the city is a hub for manufacturing textiles, food processing, fertilizers, pesticides, and industrial chemicals. The majority of the population is engaged in agricultural, commercial, financial, and industrial activities [43]. Faisalabad’s industry is greatly supported by its highly productive agricultural sector. There are roughly 1.5 million hectares of farmland in the city, the majority of which is used to grow cotton, sugar cane, and wheat. The yield of wheat is higher than the national average, averaging 40–45 maunds per acre. Faisalabad is one of Pakistan’s top regions for sugarcane production, which yields 600–700 maunds per acre, and cotton production, which yields 20–25 maunds per acre and serves as a vital source of raw materials for the textile sector. Due to their traditional agricultural background, the majority of farmers lack of knowledge about contemporary techniques of commercial farming, resulting in a subsistence-based farming system that is significantly susceptible to the negative effects of natural calamities [44]. The industrial labour force may be classified as skilled, semi-skilled, and unskilled workers, representing a socio-economically disadvantaged segment of society. The total societal welfare of the working class is rather insignificant. The area is equipped with a dry port, which may be classified as a third-generation port. This dry port plays a significant role in facilitating the transit of both raw materials and finished commodities. The emergence of the third-generation of ports occurred in the 1980s, coinciding with the global spread of containerization and the increasing demands of international commerce. Third-generation ports enhance the economic worth of products that pass through them. SEZs are a strategic tool that enables the augmentation of value-added activities inside a third-generation port [45–48]. The area selected for this study was the Allama Iqbal Special Economic Zone in district Faisalabad which is considered to be the third largest city in Pakistan by population (9.08 million). This zone is located along the right side of the Pindi Bhattian–Faisalabad section of the M4 motorway (Faisalabad–Multan Motorway) and is situated opposite the already established M3 Industrial City, which is located along the left side of the Pindi Bhattian–Faisalabad section of the M4 Motorway. Both of these zones are located between the Sahianwala and Deputywala Interchanges on the Pindi Bhattian-Faisalabad section of the M4 Motorway. This SEZ is proposed to consist of 4500 acres of land and has been declared the largest and first inaugurated SEZ in Pakistan under CPEC.

### 3.2. Data

This study relied on primary survey data collected from April 2021 to June 2021 using a semi-structured questionnaire survey. Keeping in view the information from the literature, a questionnaire was prepared to collect the data about SEZ’s impact on the socioeconomics of the indigenous people in the area. A sample of 200 respondents from the four villages was selected using simple random sampling. In the first stage, a comprehensive list of farmers for the control and treatment groups was created. In the second stage, simple random sampling was used to select the required number of selected samples and conduct face-to-face interviews in four different villages in the study area. The whole sample of 200 respondents was separated into two groups (100 each): those who sold their agricultural land and those who did not sell their land (Table 1).

**Table 1:** Sample size.

Categories	Sample Size	%
Group 1: Sale Land to SEZ	100	50
Group 1: Not Sale Land to SEZ	100	50
**Total**	200	100

The study was approved by the Qualification Examination Committee (an institutional Research Ethics Committee to ensure research quality and ethics) in the school before it began. During the data collection, the enumerators (first author and local contact persons) only interviewed workers aged greater than 18 years. No minor was involved in any activity of this research. A verbal consent of the participants was obtained before starting the interview. The following statement was written on first page of questionnaire to satisfy the participants that this study is purely for academic purpose. “All the information collected through this data collection tool will be kept highly confidential and will be used purely for academic purposes. Respondents’ identity, comments, suggestions and personal information will not be disclosed at any point of time. You are requested to participate in this important study by your consent and there is no pressure on any person to participate in this study.”

#### 3.2.1. Sampling size

This study used a 95% confidence level and a 7% margin of error for reliable and representative sample size for the target population at the village level. The following formula is used to calculate the sample size in terms of the number of respondents:

$$n=\frac{z^{2}*p*(1-p)}{e^{2}}=196$$ (1)

Where n is the target sample size, z is the 95% confidence level critical value (1.96), P is the expected proportion of the target variable (50%) and e is the margin of error (7%). To get an optimal sample size, the above-computed sample size will be adjusted to account for the finite population. The final sample size n is computed as follows:

$$n=\frac{n_{0}}{1+\frac{n_{0}}{N}}=200$$ (2)

Where n_0_ is the initial sample size calculation and N is the target population size.

#### 3.2.2. Survey instrument

The questionnaire designed for this purpose was quantitative in nature and comprised of household socioeconomic indicators including household size, education, health, employment, consumption, income, saving and domestic assets. The questionnaire was designed very carefully, so it may not exclude any details regarding the study topic. A pilot survey was done before the detailed field survey to learn about the basic knowledge of the study area and to overcome the shortcomings faced during the field survey. For this purpose, enumerators were given training, focusing on the key points during data collection, e.g., how to find and engage the respondent during the interview, the use of proxy questions about sensitive information, e.g., the income and expenditure information and how to get the true information, especially their feelings about the loss of their inherited land, the loss of current employment and the provision of new opportunities after the establishment of this SEZ in that area. This is a common misunderstanding.

#### 3.2.3 Content validity

The results of the pilot survey helped deeply to improve the final questionnaire, especially the questions regarding the qualitative aspect of the study. After collecting the data, the ground reality was done by selecting a few questionnaires randomly, going into the field and rechecking the collected data. This process of rechecking enhanced the validity of the data collected during the field survey.

### 3.3. Statistical analysis

The parametric tests that were utilized in the statistical analysis, include descriptive and inferential analyses. The cross-tabulation and its association with the study objectives are described using basic descriptive values. To determine the influence of SEZs on socioeconomic and local development, this study employed PCA to build the socioeconomic index for two groups: those who sold their agricultural land and those who did not sell their agricultural land. Furthermore, a two-sample independent t-test was utilized to compare the intervention results between the two groups. STATA v15.1 was used to examine all statistical data.

#### 3.3.1. Principal component analysis (PCA)

PCA is a standard tool in modern data analysis—in diverse fields from neuroscience to computer graphics—because it is a simple method for extracting relevant information from confusing data sets. With minimal effort, PCA provides a roadmap for how to reduce a complex data set to a lower dimension to reveal the sometimes hidden, simplified structures that often underlie it [49–51]. PCA has been validated as a method to describe SES differentiation within a population [52–54]. The mathematical form of the PCA is as follows:

$$PC_{1}=\alpha_{11}Y_{1}+\alpha_{12}Y_{2}+---+\alpha_{1}nY_{n}$$

$$PC_{2}=\alpha_{21}Y_{1}+\alpha_{22}Y_{2}+---+\alpha_{2}nY_{n}$$

−−−−−−−−−−−−−−−−−−

$$PC_{m}=\alpha_{m1}Y_{1}+\alpha_{m2}Y_{2}+---+\alpha_{m}nY_{n}$$ (3)

Where PC_1_, PC_2_ and PC_m_ are principal component equations with Yn different variables and mn equations weights. Formally, the linear combination of the SES for household i is calculated based on the following equation:

$$Y_{i}=\alpha_{1}(\frac{X_{1}-{\bar{X}}_{1}}{S_{1}})+\alpha_{2}(\frac{X_{2}+{\bar{X}}_{2}}{S_{2}})+............+\alpha_{K}(\frac{X_{K}+{\bar{X}}_{K}}{S_{K}})$$ (4)

Where yi is the SES, X_K_ is the mean of assets and other social indicators; S_K_ is the standard deviation of assets and other social indicators; and *α*_*k*_ the weights for the assets and other social indicators.

#### 3.3.2. Two sample independent t-test

The two-sample independent t-test analyses the means of two independent groups to see if there is statistical evidence that the related population means differ significantly. When the two independent samples are assumed to come from populations with similar variances, the test statistic t is calculated as follows:

$$t=\frac{\left({\bar{x}}_{1}-{\bar{x}}_{2}\right)}{\sqrt{\frac{s_{1}^{2}}{n_{1}}+\frac{s_{2}^{2}}{n_{2}}}}$$ (5)

Where:

- ${\bar{x}}_{1}$ and ${\bar{x}}_{2}$ are the sample means of the two groups,
- $s_{1}^{2}$ and $s_{2}^{2}$ are the sample variances of the two groups,
- *n*_1_ and *n*_2_ are the sample sizes of the two groups.

According to the theory of change, providing monetary compensation to farmers for land acquisition by SEZs may lead to increased financial stability, better access to education and healthcare, and overall improved quality of life. On the other side, reduce agricultural output, displace communities, and create socio-economic disparities. SEZs are essential components that, by fostering an atmosphere that is conducive to investment, innovation, job creation, and industrial development, can set off a series of positive transitions inside economies. Nonetheless, a number of variables, including as favorable legislation, the construction of infrastructure, efficient governance, and integration with more comprehensive economic plans, affect SEZ performance. In order to properly address the research objectives, this study required a rigorous alignment of statistical approaches in order to evaluate the impact of SEZs on the livelihood of residents who have sold their lands for SEZ activity. The main justification for choosing PCA and the two-sample independent t-test was that they were appropriate and pertinent for analyzing the effects on local and socioeconomic development [53]. Furthermore, the use of PCA in this particular setting was supported by earlier research that validated the method’s ability to distinguish socioeconomic differences within populations [52–54]. In order to identify statistically significant differences in means between two different groups: those who sold their agricultural land and those who kept it. The two-sample independent t-test was also strategically used. These statistical techniques were modified because of their innate capacity to respond to the particular study questions.

## 4. Results

### 4.1. Descriptive analysis

This survey study involved 200 respondents who answered a set of questions includes 100 for those who sold his land for SEZ and 100 are those who did not sale Agri-land and continuing cultivation. These participants were further divided into certain characteristics like age, education, job, and how their household earns money. Age was split into four groups: 20–40, 41–60, 61–80, and above 80. In the oldest age group (above 80), there was only one person who sold their agricultural land, and none who didn’t sell any land. Education was divided into six categories: illiterate, primary, middle, matriculation, intermediate, graduation, and masters or higher. This distribution showed a normal spread, with fewer people at the extreme ends. Both those who sold their agricultural land and those who didn’t were included in the study. Regarding the primary source of income, there were various responses, which have been sorted and listed. The most common titles were "wage-employed agriculture" and "self-employed agriculture." Since the area studied relies heavily on agriculture, many people are directly or indirectly involved in this sector. (Table A1 in S1 Appendix).

The establishment of SEZs has impacted land values and rents. The land was acquired in two phases, first in 2005 and then in 2020. According to all 200 respondents, land prices have risen since the introduction of SEZs. Nearly an equal number of respondents mentioned that prices have increased by 2 to 5 times. Similarly, all 200 participants indicated that land rents have escalated, with varying rates of increase. The government of Pakistan paid an average price of Rs.2 million. Following the introduction of SEZs, respondents reported that the rate of agricultural land has increased to between Rs.4 to 6 million per acre. The confidence of responses from individuals within the respondent group who do not sell their land was similar to that of farmers who sell their agricultural land. On the other hand, the average Government Deputy Commissioner’s office rate (DC rate) for the link, off, and main road prices of agricultural land in Chak No.152 RB, Chak No.154 RB, and Chak No. 144 RB ranges between Rs.3.5 million to Rs.4 million per acre [55].

Regarding the land used for SEZ establishment, most respondents noted that both agricultural and undeveloped land were utilized for this purpose. The creation of SEZs has led to an increase in the value of all types of land uses, including open, agricultural, residential, commercial, and industrial areas. However, none of these land uses showed an exponential surge in their value during this period (Table A2 in S1 Appendix).

More than 85% of our respondents from both groups claimed that before SEZ, the daily wage rate was 400 PKR/day, which has increased to up to 800 PKR/day. The establishment of SEZ has increased the workforce in the area and this force belongs to different categories, i.e. general, specialized and skilled labour, supervisory and executive levels. General and skilled labour claimed to have a greater increase in the workforce as compared to other categories after the study area’s SEZ. When it was asked which type of business had increased more after SEZ, 31% of those who sold their agricultural land and 35% of those who did not sold their agricultural land gave their opinion that it was property and real estate. Another business type that got a prominent response was the "transportation of goods". Almost equal response (30%) was noted from both groups for the business type "provision of raw materials". There was zero response to the business types "retail" and "wholesale". An increase in industry type was the next question that was asked of the respondents. Large-scale and heavy industry were the two types that got the most prominent and almost equal response (38% to 40%) from both the groups. The cottage industry was the only industry type that was claimed not to have been established or increased in the area since SEZ (Table A3 in S1 Appendix).

Questions were asked about inward and outward migration from the area, the source areas of migration and the resettlement buffer of inward migrants in the area. During this survey, the positive and negative effects of SEZ on the demography and social life of residents were also considered. Inward and outward Migration: Almost 85% of respondents in both groups reported inward migration following the announcement of an SEZ in the area. This migration has been done by the people of central and south Punjab and Sindh provinces. As these areas are not very rich in agricultural, industrial, and mineral resources, people are facing unemployment and poverty on a large scale, hence forcing this population towards big cities and industrial and commercial hubs for job searching. People from other parts of the country, e.g., South Sindh, Balochistan, Khyber Pakhtunkhwa, Northern areas (Gilgit-Baltistan), Azad Kashmir, and North Punjab, are not attracted to this area for work due to long distances and social-cultural differences. These migrants tend to settle in areas near the reach of the SEZ. From both groups, 34% of the population claimed that a 2-kilometre radius of the SEZ was the best circle to describe the resettlement of these inward migrants. The SEZ has both positive and negative impacts on the social life of the residents in the area. Positive effects include the transfer of knowledge and increment in the workforce, while negative effects include the increase in house rents and crimes. Almost 18.5% of respondents in both groups believe that SEZ has no positive effect on the study area. More than half of the people living in the study area claimed that house rents had increased due to the increased demand for housing after the establishment of SEZ in the area. 37% of those who sold their agricultural land and 42% of those who did not sold their agricultural land claimed that there had been an increase in crime rates after the SEZ. About 17% of both groups stated that this SEZ has no negative effect on the social and cultural life of inhabitants in the study area (Tables A4-1-A4-3 in S1 Appendix).

Almost 50% of the participants from each group think that the establishment of SEZ has destroyed the agricultural farmlands in the area. Respondents also stated that SEZ has destroyed the scenic beauty and biodiversity of the area. About 50% of respondents from both groups stated that this SEZ has brought air pollution to the area in its initial phase, and they expect the situation to become worse when industries are fully operationalized. About 30% claimed that there is water contamination due to industrial wastewater in the area, while 23% of respondents complained about the solid waste that has been spread since the inception of SEZ. The SEZ has caused the spread of diseases, especially respiratory and water-borne diseases, in the area. According to community perception, SEZ has caused respiratory diseases such as cough, flu, and lung disease, while contaminated water is causing diarrhea, hepatitis, etc. in the area. The decision to establish the SEZ in the area has caused traffic problems as well. Heavy machinery use has increased rapidly, which not only causes traffic jams on narrow roads but also becomes a cause of road accidents. Respondents from both groups believe that heavy traffic on roads, traffic jams, and road accidents have increased due to this SEZ (Table A5 in S1 Appendix). 59% of respondents think that there is no technology transfer associated with the establishment of SEZ in the study area. Those who claim that SEZs have brought technology transfer to the area have differing opinions when it comes to which sector they are referring to. According to the respondents, the agricultural sector is the most affected. The industrial sector and skilled labour are the main sectors where technology has been transferred. In the opinion of the respondents, the commercial sector could not get a prominent transfer of technology (Table A6 in S1 Appendix).

### 4.2. Principal component analysis results

The socioeconomic score (SES) of the study area households was constructed using principal component analysis (PCA), which is a proxy of household well-being. It is commonly used in social science to build scores and reduce the number of variables into simple index scores. Principal components are new variables that are created by combining or mixing the initial variables linearly. The new variables are uncorrelated because of these combinations, and the majority of the information from the initial variables is compressed into the first components [54].

PCA tries to put as much information as possible in the first component, then as little information as possible in the second, and so on. The first nine components, along with more than one eigenvalue, accounted for a 65% of the total variation. The first component accounted for 26% of the total, followed by the second (10%), third (10%), fourth (10%), and so on. Furthermore, the eigenvalue represents the component’s variance and is considered critical for values greater than one. All the variables’ values add up to the total number of variables. The size of one component’s eigenvalue compared to the next component’s eigenvalue is the difference. Thus, the first nine principal components with eigenvalues greater than one explained 65% of the data. (Tables A7 and A8 in S1 Appendix).

**Fig 1:**
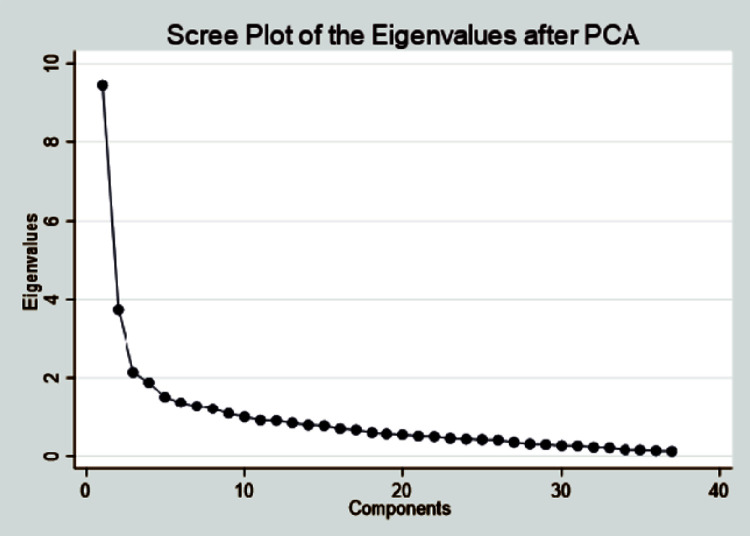
Scree plot of the eigenvalues after PCA.

A scree plot is a line plot of the eigenvalues of factors or principal components in an analysis. The scree plot is used to determine the number of factors to retain in exploratory factor analysis or principal components to keep in principal component analysis. The first nine components with more than one eigenvalue are explained in Fig 1.

### 4.3. Distribution of SES data

**Fig 2:**
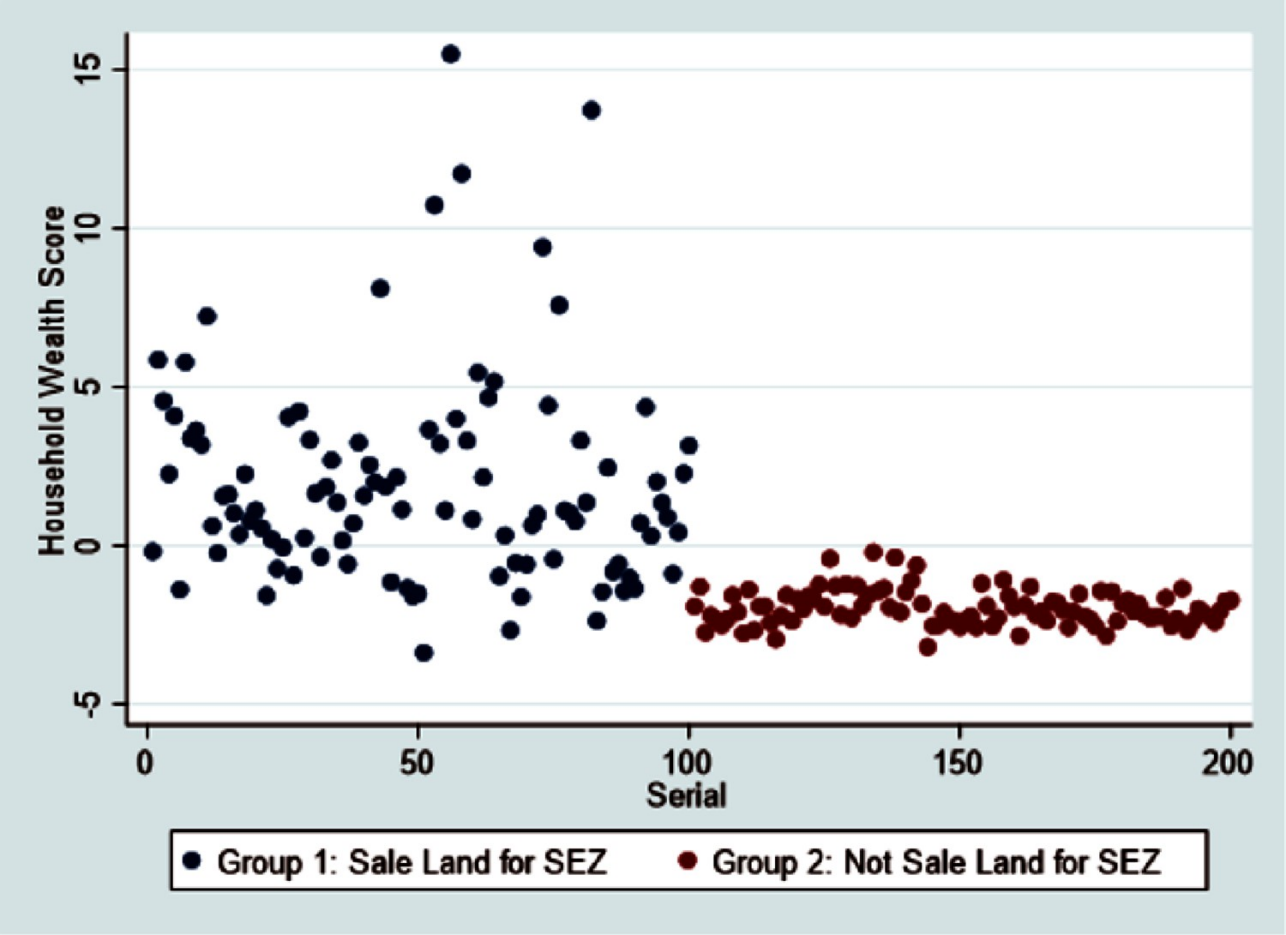
Distribution of SES data in the study area.

Fig 2 depicts the SES distribution of two study groups: those who sell their agricultural land for SEZ and those who do not sell their agricultural land for SEZ. There is a statistically significant difference between the two groups. In a comparison between two groups, the treatment group that received Pakistani rupees had a higher SES. It is clear that households who sell their land invest the proceeds in asset-generating activities, whereas the agriculture sector in Pakistan faces numerous challenges in terms of productivity, efficiency, and profitability. In comparison to households engaged in non-agricultural and agricultural activities, households engaged in agriculture have a lower SES.

The mean, standard deviation, minimum, and maximum values of SES were displayed in Table 1, calculated from household assets and living standards using PCA technique. The average score for those who sell their agricultural land for SEZ is 1.95, with a standard deviation of 3.32 and a range of -3.38 to 15.50. Those households, who did not sell their agricultural land for SEZ, had an average score of -1.96, with a range of -3.19 to -0.21 and a standard deviation of 0.56. The SES analysis indicates that households who do not sell their agricultural land are worse off in terms of SES than those who do. This is because they have fewer assets.

### 4.4. Inferential analysis of impact of SEZs on socioeconomic status

Determining the well-being of households through SEZs is more a matter for political decision-makers and offers the federal and provincial governments’ support for the future development of SEZs and their economy. Various methods are available to assess the impact of any programme and intervention, including experimental and non-experimental designs. In this study, the simplest method was used to calculate the effect of the mean SEZ of the programme location. This was done with a two-sample independent t-test with probability.

The SES result shows that the difference between those who sell their agricultural land and those who did not sell their agricultural land is significant if the other factors remain constant. That is, the placement of the SEZ programme in Pakistan will initially improve the economic situation of the households. The households that sold their agricultural land received a significant amount of cash. The impact of this cash has significantly changed the economic situation of those who sold their agricultural land compared to those who did not sell their agricultural land. It was found by [56] that SEZ compensation payments in India were used for asset purchases, satisfying social commitments before the situation worsened marriage of a daughter or son, debt repayment, basic health care and medicine, and everyday household basic needs. The same trend was observed in our case. The average score of the SES was estimated at -1.96 for those who did not sell their agricultural land and 1.95 for those who sold their agricultural land. A statistically -3.92 difference was estimated between these two groups and it is significant at 1%. (Table 2).

**Table 2:** Household SES.

	N	Mean	Std. Err.	[95% Conf. Interval]		P-value
Group 1 Not Sale Land for SEZ	100	-1.96	0.06	-2.08	-1.85	.000*
Group 2 Sale Land for SEZ	100	1.95	0.33	1.29	2.61	
Combined	200	-0.01	0.22	-0.44	0.42	
Difference		-3.92	0.34	-4.58	-3.25	

The impact of per capita education spending was recorded at Rs. 9029 per capita per month for those who did not sell their agricultural land and Rs. 8575 for those who did. The difference in per capita education spending between the two groups was Rs. 454, which is statistically negligible. It suggests that the total and general per capita expenditure on education was almost equal between the two categories (Table 3). As [56] mentioned, education was not among the top five priority areas where this compensation amount was spent. Previous research, e.g [13] also found that high school dropout rates have increased in nearby SEZ districts, suggesting that working in SEZs is an appealing alternative to staying in school for young people.

**Table 3:** Per capita education expenditure.

	N	Mean	Std. Err.	[95% Conf. Interval]		P-value
Group 1 Not Sale Land for SEZ	100	9029.3	365.1	8304.9	9753.7	0.2344
Group 2 Sale Land for SEZ	100	8575.0	508.4	7566.4	9583.7	
Combined	200	8802.2	312.6	8185.8	9418.5	
Difference		454.2	625.9	-780.0	1688.4	

Furthermore, the impact of per capita health spending was recorded at an average of Rs. 5,251 per capita per month for those who did not sell their agricultural land and Rs. 6,554 for those who did. The difference in the two-group per capita health spending was Rs. 1,303, which is statistically significant at 1%. It means that total and general per capita health spending grew or increased more between those who sold their agricultural property and those who did not (Table 4). The same trend was noted by [43]. A much higher percentage of compensation (21%) was used to cover various types of consumption, such as daily necessities, medical care, and wedding expenses.

**Table 4:** Per capita health expenditure.

	N	Mean	Std. Err.	[95% Conf. Interval]		P-value
Group 1 Not Sale Land for SEZ	100	5,251.2	340.2	4,576.2	5,926.1	0.012*
Group 2 Sale Land for SEZ	100	6,554.9	461.2	5,639.8	7,470.0	
Combined	200	5,903.0	289.5	5,332.1	6,474.0	
Difference		-1,303.7	573.1	-2,433.8	-173.6	

Similarly, to the previous results, the impact of per capita saving was recorded at an average of Rs. 16, 672 per capita per month for those who did not sell their agricultural land and Rs. 19, 843 for those who did. The difference in per capita savings between the two groups was Rs. 3,171, which is statistically significant at 1%. It means that the total and general per capita savings grew or increased between those who sold and those who did not sell the agricultural property (Table 5). These results slightly differ from those [56] who discovered that persons who received compensation were able to invest little usefully for the future and were obliged to settle debts or meet continuous costs while unemployed. The main reason for this difference is that there were very few scheduled castes and marginal groups existed in the areas where SEZs were started in India, while in our case, the Allama Iqbal SEZ was initiated in an area where the majority of the population was from "zameendaar" (landowners who are high caste in social set up of society) background, who were not too poor as compared to Indian SEZ, thus they had minor social pressure from moneylenders to settle the debts.

**Table 5:** Per capita saving.

	N	Mean	Std. Err.	[95% Conf. Interval]		P-value
Group 1 Not Sale Land for SEZ	100	16,672.3	845.333	14,995	18,349.7	0.0169*
Group 2 Sale Land for SEZ	100	19,843.5	1,218.75	17,425.2	22,261.7	
Combined	200	18,257.9	748.236	16,782.4	19,733.4	
Difference		-3,171.1	1,483.22	-6,096.1	-246.2	

## 5. Discussion

### 5.1. Influence of monetary compensation on farmers’ well-being

The characteristics of the respondents suggest that the average age observed is in the range of 41 to 60 years and includes the majority of people in both classes. In the education sector there is a typical distribution with a relatively small number of respondents at the extremes. The main source of income was agriculture, with the population in the study area working directly or indirectly in the agricultural sector. Individuals work either on their own land or as wage laborers on other people’s agricultural lands. Similar observations were made by [56, 57], showing that many people who lost their land became farmers with smaller landholdings, while others became landless. The results showed that the compensation amount for the acquired land not only improved the socio-economic living conditions of the indigenous population in the short term, but also changed their form of employment from agriculture to labor work. This change was accompanied by higher health spending and greater prosperity for private households. Minor changes were noted in education expenditure and new houses were built, often for purposes such as child marriage, vehicle purchases and dowry expenditure related to weddings within the Special Economic Zone (SEZ). However, such spending is typically unproductive and can lead to long-term economic disadvantage rather than sustainable growth. Previous studies have largely focused on geopolitics [57].

### 5.2. Effect of SEZs’ land acquisition on local development of indigenous farmers

Under the “self-employed farming” label, a significant proportion of households– 68 out of 100 respondents–did not sell their land. This makes sense because this is the group from which the government has not acquired the agricultural land and they tend to continue their work. The development of the special economic zone has also affected property values and property rents. The government acquired the land twice, once in 2005 and again in 2020. The majority of our respondents are those whose land was acquired in 2020. All 200 respondents stated that land prices and land rent have increased since the introduction of the special economic zone in this area [56]. This was because the property values in the hamlet following the special economic zone were significantly higher than the compensation that the authorities granted to those who lost the property. According to the findings of [15], rising local labour demand boosts both commercial and residential demand for land, raising the price of existing houses and structures. Even with this increase in developed land supply, the price of the existing property would stay permanently higher, because existing land has certain locational benefits over newly created land. It is demonstrated in [13] that, in comparison to other districts, land prices in SEZ areas tend to improve while wage levels stay essentially stable. When it was asked how many times the land rate had increased, the response was a blend of answers. Almost an equal number of respondents stated that it had increased by 2 to 5 times.

Despite the relative abundance of labor in developing nations, certain SEZs, particularly those situated in metropolitan areas, encounter difficulties in attracting and retaining personnel. For instance, SEZs in Malaysia and Mauritius have faced labor shortages, necessitating the importation of foreign workers [58]. SEZs and industrial clusters are perceived to attract Foreign Direct Investment (FDI) due to the advantages of location that outweigh policy distortions. Research modeling the effects of SEZs and clusters on FDI supports this notion [59]. Similarly, our findings suggest that the SEZ under study is poised to attract both local and foreign investments in the future. Increased FDI is associated with improvements in the business climate within these zones. However, empirical evidence regarding SEZs’ effects on technological spillovers remains scant, largely due to the underdevelopment of the domestic supply chain [60].

### 5.3. Socioeconomic impact of SEZs on the farming community

When talking about the land use used to establish the special economic zone, nearly 40% of respondents said it was agricultural land, while about 60% felt it was a mix of agricultural and undeveloped land areas traded. Other studies e.g. [56] also mentioned that the loss of arable land in the areas affected by the SEZ project has led to a significant decline in agriculture. The response to the increase in land value of different land uses is almost balanced, and none of the highlighted land uses experienced an exponential increase in value during this period. According to the findings of [15], the increasing demand for local labor increases both commercial and residential demand for land and increases the price of existing houses and structures. Even with this increase in developed land supply, the price of the existing property would stay permanently higher, because existing land has certain locational benefits over newly created land. It is demonstrated in [13] that, in comparison to other districts, land prices in SEZ areas tend to improve while wage levels stay essentially stable. When it was asked how many times the land rate had increased, the response was a blend of answers. Almost an equal number of respondents stated that it had increased by 2 to 5 times.

SEZ has also affected economic activities. More than 85% of the respondents from both groups claimed that daily wage rates have increased (almost doubled) since the inception of SEZ in this area. The same was found by [9], the income level and earning ability of workers within the region have been one of the most significant economic repercussions of SEZs on the local economy [15]. Studies found that increased local labour demand is likely to reduce local unemployment and put upward pressure on real wages in the short run. Some studies e.g. [60] found that in SEZs, salaries for unskilled workers are often lower at first, then rise over time. It was also mentioned by [13] that in comparison to other districts in Cambodia, pay levels in SEZ zones are virtually stable. The reason for this is that SEZ companies tend to recruit mostly female employees, who have few other choices in the formal sector.

Therefore, in Cambodia, evidence of modest employment and wage impacts in treated areas is consistent with previous research concentrating on SEZ effects at the company level [61, 62]. SEZ also has increased the workforce in the area as mentioned by [7]. Studies e.g. [58, 63] found that SEZs in China resulted in the concentration of human capital and an increase in total factor productivity (TFP). However, the evidence is not conclusive on employment in the local economy and general and skilled labour are claimed to have a greater increase in the workforce as compared to other categories. This increase in the workforce can be seen as a proxy of the increase in employment in the area as an impact of the SEZ. Overall evidence on employment effects of SEZs is mixed in the literature,—some studies as reviewed by [64], failed to find any effects such as [65], while others report significant increases in employment rates as a result of the extension of the SEZ programme in the United States [16], but [60] disagrees with them arguing that this rise in employment and income is simply because these are relatively poor rural and urban regions where employees are granted tax credits and block grants to use for investments, training, and housing. Therefore, there is no significant flow of labour into these zones. Studies that look at the benefits of policies that encourage businesses to recruit people from underserved regions indicate that they have a considerable beneficial impact on employment [66, 67]. It was claimed by [13] that SEZs increase female employment but have a minor impact on the aggregate formal employment share. But sometimes the situation is different.

### 5.4. Impact on business and industry types

Inquiries about the business landscape post-SEZ implementation yielded varied responses. Approximately 30–35% of respondents identified the "property and real estate business" as experiencing growth. This trend is often skewed towards benefiting upper-income groups, contributing to greater income disparities between landowners and landless individuals [15]. Another notable business sector that saw expansion was "transportation of goods," garnering a 35–40% response. Approximately 30% of respondents also noted growth in the "provision of raw materials" sector. Conversely, there was minimal to no reported growth in "Retail" and "wholesale" businesses. SEZs have facilitated opportunities for both local and foreign direct investment, impacting wages, productivity, and exports, as highlighted in previous studies [9].

However, it was concluded by [68] that the attraction of FDI to SEZ areas is primarily driven by low wage rates. For example, monthly wages in Ethiopia range from $40 to $60, significantly lower than those in countries like China, making it an attractive prospect for foreign investors [60]. In terms of industry types, large-scale and heavy industries received the most prominent responses, ranging from 38% to 40% from both respondent groups. Conversely, small-scale and hi-tech industries received minimal responses, approximately 11–12% and 9%, respectively, from both groups. The cottage industry, focused on small-scale production, was notably absent in terms of establishment or growth within the SEZ area, aligning with the SEZ’s strategic focus and the government’s separate initiatives through the Small and Medium Enterprise Development Authority (SMEDA).

### 5.5. Demographic and social impacts

The establishment of the SEZ has significantly influenced demographic patterns, particularly through inward migration from neighboring provinces such as central and southern Punjab and Sindh. This influx underscores the spillover effects of the SEZ on regional demographics and socioeconomic dynamics. Indirect spillover effects on non-SEZ regions have been observed, including impacts on female employment and changes in high school dropout rates [13]. Economic factors such as unemployment and poverty in source regions (discussed by [53, 69]) drive migration towards urban and industrial centers in search of employment opportunities. This migration pattern, described by [57] as a form of artificially induced urbanization common in developing countries, results in settlement near SEZs, particularly within a radius of 1–3 kilometers.

### 5.6. Social and environmental concerns

The SEZ has brought about both positive and negative impacts on the social fabric of the study area. Positively, there has been a transfer of knowledge and an increase in the labor force due to employment opportunities generated by the SEZ [57]. However, negative repercussions include rising house rents and increased crime rates [56]. Environmental concerns are prevalent among the local community, with perceptions of increased air and water pollution, solid waste generation, and associated health risks such as respiratory and waterborne diseases. Approximately 50% of respondents believe that the establishment of the SEZ has led to the destruction of agricultural lands, impacting biodiversity and scenic beauty [56]. Concerns about polluted air causing respiratory ailments and contaminated water leading to diseases like diarrhea and hepatitis are also widespread, affecting approximately 60% of respondents.

## 6. Conclusions and policy implications

The monetary compensation received by farmers from the acquisition of their land for the SEZ has significantly impacted their well-being. Although the compensation was provided at government rates, many farmers were dissatisfied with the amount received. The utilization of compensation varied among villagers, affecting their well-being in several ways. The compensation led to notable changes in property and asset ownership. Villagers invested in various assets such as land parcels, dowries, vehicles, shops, houses, and livestock. This shift in asset ownership suggests a diversification of their economic base. Some villagers saved the compensation in banks, which might provide financial security in the long term. Investments in domestic assets like houses and home appliances (cars, televisions, refrigerators) indicate an improvement in the quality of life and social status. The nature of work for many villagers changed from farming to labor in the SEZ. This shift may have implications for their long-term economic stability and well-being. Many households did not invest wisely in income-generating activities, leading to concerns about the sustainability of their improved socioeconomic status. The lack of basic vocational, entrepreneurial, and financial training has limited the villagers’ ability to make sustainable investments.

The establishment of the SEZ and the acquisition of agricultural land from indigenous farmers have led to mixed effects on local development: While the compensation provided a temporary financial boost, the lack of investment in income-generating activities means the economic development may not be sustainable. These households did not spend their money on basic businesses opportunities such as the purchase of agricultural land and livestock, trading, and other commercial operations, their socioeconomic situation may not be sustainable in the long run. The transition from farming to labor work in the SEZ represents a significant change in the local economy. This shift could provide new opportunities but also poses risks if the jobs are not stable or well-paying. The conversion of agricultural land to industrial use has reduced the available farmland, potentially impacting local food production. Increased air and water pollution and disturbances to wildlife and biodiversity have negative implications for local development and health. The presence of the SEZ could lead to improved infrastructure and services in the long term. The government should compensate for environmental degradation externalities in the form of public parks and District Headquarters (DHQ) level hospitals. From a policy perspective, SEZs may be designed and operated following the United Nations’ Sustainable Development Goals (SDGs), particularly focusing on goals no. 5, 7, 8, 9, 10, and 12.

The socioeconomic impact of the SEZ on the farming community includes both positive and negative aspects: The compensation has enabled many households to acquire new assets and improve their living standards. The SEZ has created new job opportunities, although these are primarily labor-oriented positions. The lack of investment in sustainable income-generating activities means that the initial economic benefits may not last. Increased pollution and associated health issues present significant challenges to the well-being of the farming community. Government and private organizations should provide training to help villagers invest in sustainable businesses. Efforts to raise awareness about income-generating activities and financial management are essential for long-term socioeconomic development. Addressing pollution and health issues is crucial to ensure the overall well-being of the community.

## Supporting information

S1 File This file contains all data relevant to field survey and analysis. (XLS)

S1 Appendix This file contains all the appendices of the manuscript. (DOCX)
