# Supplementary material for: Impact of special economic zones on socioeconomics and local development in Pakistan: Evidence from Allama Iqbal Special Economic Zone, Faisalabad
Source: PLoS One. 2024 Nov 14;19(11):e0310488. doi: 10.1371/journal.pone.0310488 (PMC11563473; doi:10.1371/journal.pone.0310488)
Supplement: S1 Appendix — (DOCX) [file pone.0310488.s002.docx]

**Appendix-A**

**Table: A1. Respondent’s Characteristics**

|  |  | Treatment Group | | Control Group | | Overall Sample | |
| --- | --- | --- | --- | --- | --- | --- | --- |
|  |  | **N** | **%** | **N** | **%** | **N** | **%** |
| **Gender** | **Male** | 100 | 100.0 | 100 | 100.0 | 200 | 100.0 |
| **Age** | **20-40** | 18 | 23.0 | 28 | 28.0 | 46 | 23.0 |
|  | **41-60** | 56 | 57.5 | 59 | 59.0 | 115 | 57.5 |
|  | **61-80** | 25 | 19.0 | 13 | 13.0 | 38 | 19.0 |
|  | **81 and above** | 1 | 0.5 | 0 | 0.0 | 1 | 0.5 |
| **Education** | **Illiterate** | 9 | 9.0 | 15 | 15.0 | 24 | 12.0 |
|  | **Primary** | 2 | 2.0 | 12 | 12.0 | 14 | 7.0 |
|  | **Middle** | 35 | 35.0 | 27 | 27.0 | 62 | 31.0 |
|  | **Matriculation** | 28 | 28.0 | 27 | 27.0 | 55 | 27.5 |
|  | **Intermediate** | 15 | 15.0 | 12 | 12.0 | 27 | 13.5 |
|  | **Graduation** | 6 | 6.0 | 5 | 5.0 | 11 | 5.5 |
|  | **Masters** | 5 | 5.0 | 2 | 2.0 | 7 | 3.5 |
| **Occupation/ Primary Sources of Income** | **Salaried (Private/Government)** | 9 | 9.0 | 13 | 13.0 | 22 | 11.0 |
|  | **Pension holder** | 4 | 4.0 | 5 | 5.0 | 9 | 4.5 |
|  | **Wage employed- agricultural** | 38 | 38.0 | 0 | 0.0 | 38 | 19.0 |
|  | **Wage employed- non-agricultural** | 10 | 10.0 | 0 | 0.0 | 10 | 5.0 |
|  | **Self-employed- business** | 11 | 11.0 | 14 | 14.0 | 25 | 12.5 |
|  | **Self-employed- agriculture** | 20 | 20.0 | 68 | 68.0 | 88 | 44.0 |
|  | **Self-employed- construction and major r** | 6 | 6.0 | 0 | 0.0 | 6 | 3.0 |
|  | **Unemployed-No work available** | 2 | 2.0 | 0 | 0.0 | 2 | 1.0 |

**Table: A2. Impact of SEZ on Land**

|  |  | Treatment Group | | Control Group | | Overall Sample | |
| --- | --- | --- | --- | --- | --- | --- | --- |
|  |  | N | % | N | % | N | % |
| **Land acquire** | Yes | 100 | 100 | 0 | 0 | 100 | 50 |
|  | No | 0 | 0.0 | 100 | 100 | 100 | 50 |
| **Time of land acquire** | 0 | 0 | 0.0 | 0 | 0 | 100 | 50 |
|  | 2005 | 13 | 13 | 0 | 0 | 13 | 6.5 |
|  | 2020 | 87 | 87 | 0 | 0 | 87 | 43.5 |
| **Increase in land rate** | Increase in land rate | 100 | 100 | 100 | 100 | 200 | 100 |
|  | No change | 0 | 0.0 | 0 | 0 | 0 | 0 |
| **Increment in land rate** | <50% | 1 | 1.0 | 7 | 7.0 | 8 | 4.0 |
|  | 51-100% | 15 | 15.0 | 20 | 20.0 | 35 | 17.5 |
|  | 101-150% | 25 | 25.0 | 28 | 28.0 | 53 | 26.5 |
|  | 151-200% | 33 | 33.0 | 24 | 24.0 | 57 | 28.5 |
|  | >200% | 26 | 26.0 | 21 | 21.0 | 47 | 23.5 |
| **Increase in land rent** | Increase in land rent | 100 | 100 | 100 | 100.0 | 200 | 100 |
|  | No change | 0 | 0.0 | 0 | 0 | 0 | 0 |
| **% of the increase in land rent** | <50% | 2 | 2.0 | 5 | 5.0 | 7 | 3.5 |
|  | 51-100% | 20 | 20.0 | 18 | 18.0 | 38 | 19.0 |
|  | 101-150% | 29 | 29.0 | 33 | 33.0 | 62 | 31.0 |
|  | 151-200% | 26 | 26.0 | 22 | 22.0 | 48 | 24.0 |
|  | >200% | 23 | 23.0 | 22 | 22.0 | 45 | 22.5 |
| **Land use** | Bare land | 0 | 0 | 0 | 0 | 0 | 0 |
|  | Agriculture land | 37 | 37.0 | 47 | 47.0 | 84 | 42.0 |
|  | both bare and agricultural land | 63 | 63.0 | 53 | 53.0 | 116 | 58.0 |
| **Increment in land value** | Increase the value of open land | 39 | 21.3 | 37 | 22.1 | 76 | 21.7 |
|  | Increase the value of agricultural land | 41 | 22.4 | 37 | 22.1 | 78 | 22.3 |
|  | Increase the value of residential land | 35 | 19.1 | 35 | 20.9 | 70 | 20.0 |
|  | Increase the value of commercial land | 32 | 17.5 | 26 | 15.6 | 58 | 16.6 |
|  | Increase the value of industrial land | 36 | 19.7 | 32 | 19.1 | 68 | 19.4 |

**Table: A3. Impact of SEZ on Economic Activity**

|  |  | Treatment Group | | Control Group | | Overall Sample | |
| --- | --- | --- | --- | --- | --- | --- | --- |
|  |  | **N** | **%** | **N** | **%** | **N** | **%** |
| **Increase in daily wage** | **Yes** | 88 | 88.0 | 86 | 86.0 | 174 | 87.0 |
|  | **No** | 12 | 12.0 | 14 | 14.0 | 26 | 13.0 |
| **Average daily wage before SEZ** | **350** | 18 | 18.0 | 16 | 16.0 | 34 | 17.0 |
|  | **400** | 57 | 57.0 | 43 | 43.0 | 100 | 50.0 |
|  | **450** | 25 | 25.0 | 41 | 41.0 | 66 | 33.0 |
| **Current Average daily wage** | **750** | 14 | 14.0 | 15 | 15.0 | 29 | 14.5 |
|  | **800** | 47 | 47.0 | 41 | 41.0 | 88 | 44.0 |
|  | **850** | 26 | 26.0 | 29 | 29.0 | 55 | 27.5 |
|  | **900** | 13 | 13.0 | 15 | 15.0 | 28 | 14.0 |
| **Increase in labour** | **General labour** | 48 | 30.8 | 55 | 32.9 | 103 | 31.9 |
|  | **Specialized labour** | 29 | 18.6 | 29 | 17.4 | 58 | 18.0 |
|  | **Skilled labour** | 46 | 29.5 | 46 | 27.5 | 92 | 28.5 |
|  | **Supervisory Level** | 21 | 13.5 | 20 | 12.0 | 41 | 12.7 |
|  | **Executive Level** | 12 | 7.7 | 17 | 10.2 | 29 | 9.0 |
| **Increase in investment** | **More foreign investment** | 52 | 51.5 | 51 | 48.1 | 103 | 49.7 |
|  | **More local investment** | 49 | 48.5 | 55 | 51.9 | 104 | 50.2 |
| **Increase in Business** | **Property and Real Estate** | 37 | 30.1 | 37 | 34.9 | 74 | 32.3 |
|  | **Wholesale** | 0 | 0 | 0 | 0 | 0 | 0 |
|  | **Retail** | 0 | 0 | 0 | 0 | 0 | 0 |
|  | **Transportation of goods** | 48 | 39.0 | 37 | 34.9 | 85 | 37.1 |
|  | **Provision of raw material** | 38 | 30.9 | 32 | 30.2 | 70 | 30.6 |
| **Increase in Industry** | **Cottage Industry** | 0 | 0 | 0 | 0 | 0 | 0 |
|  | **Small Scale Industry** | 16 | 12.2 | 14 | 10.9 | 30 | 11.5 |
|  | **Large Scale Industry** | 53 | 40.5 | 51 | 39.5 | 104 | 40.0 |
|  | **Heavy Industry** | 50 | 38.2 | 52 | 40.3 | 102 | 39.2 |
|  | **Hi-tech Industry** | 12 | 9.2 | 12 | 9.3 | 24 | 9.2 |

**Table: A 4-1. Impact of SEZ on demography**

|  |  | Treatment Group | | Control Group | | Overall Sample | |
| --- | --- | --- | --- | --- | --- | --- | --- |
|  |  | N | % | N | % | N | % |
| **Inward Migration** | **Yes** | 86 | 86.0 | 84 | 84 | 170 | 85 |
|  | **No** | 14 | 14.0 | 16 | 16 | 30 | 15 |
| **Usually, come from** | **Central+South Punjab** | 15 | 15.0 | 14 | 14 | 29 | 14.5 |
|  | **Sindh+South Punjab** | 38 | 38.0 | 38 | 38 | 76 | 38 |
|  | **South Punjab** | 33 | 33.0 | 32 | 32 | 65 | 32.5 |
| **Inward Migration usually settle** | **less than 1 km** | 17 | 17.0 | 27 | 27 | 44 | 22 |
|  | **less than 2 km** | 35 | 35.0 | 33 | 33 | 68 | 34 |
|  | **less than 3 km** | 34 | 34.0 | 24 | 24 | 58 | 29 |

**Table: A 4-2. Impact of SEZ on demography**

|  | Treatment Group response | | Control Group response | | Overall Sample response | |
| --- | --- | --- | --- | --- | --- | --- |
| **Inward Migration** | **Number of persons** | **Number of Families** | **Number of persons** | **Number of Families** | **Number of persons** | **Number of Families** |
| **N** | 100 | 91 | 100 | 94 | 170 | 170 |
| **Mean** | 4432.0 | 629.7 | 3956 | 728.7 | 4934.2 | 740 |
| **SD** | 4188.6 | 488.4 | 3890.1 | 590.3 | 3941.6 | 526.1 |
| **Min** | 0 | 0 | 0 | 0 | 500 | 70 |
| **Max** | 15000 | 1900 | 17000 | 2300 | 17000 | 2300 |

**Table: A 4-3. Impact of SEZ on demography**

|  |  | Treatment Group | | Control Group | | Overall Sample | |
| --- | --- | --- | --- | --- | --- | --- | --- |
|  |  | N | Response % | N | % | N | % |
| **Positive Effects of SEZ** | **Transfer of knowledge** | 20 | 20.4 | 23 | 25.5 | 43 | 22.7 |
|  | **More workforce** | 59 | 60.2 | 48 | 53.3 | 107 | 56.6 |
|  | **No positive effect** | 18 | 19.4 | 19 | 21.1 | 37 | 20.6 |
| **Negative Effects of SEZ** | **Increased House Rents** | 48 | 47.5 | 45 | 42.4 | 93 | 44.9 |
|  | **Increase in Crimes** | 37 | 36.63 | 42 | 39.6 | 79 | 38.1 |
|  | **No Negative effect** | 16 | 15.8 | 19 | 17.9 | 35 | 16.9 |

**Table: A5. Impact of SEZ on the Environment**

|  | Treatment Group | | Control Group | | Overall Sample | |
| --- | --- | --- | --- | --- | --- | --- |
|  | N | % | N | % | N | % |
| **Destroyed scenic beauty** | 43 | 27.0 | 48 | 29.6 | 91 | 28.3 |
| **Destroyed natural farmland** | 78 | 49.1 | 80 | 49.4 | 158 | 49.2 |
| **Disturbed biodiversity** | 38 | 23.9 | 34 | 21.0 | 72 | 22.4 |
| **Air pollution** | 84 | 48.0 | 85 | 48.8 | 169 | 48.4 |
| **Water contamination** | 51 | 29.1 | 48 | 27.6 | 99 | 28.8 |
| **Solid waste** | 40 | 22.9 | 41 | 23.5 | 81 | 23.2 |
| **Respiratory and lung diseases** | 78 | 59.5 | 81 | 61.3 | 159 | 60.4 |
| **Waterborne diseases, including cholera, typhoid** | 53 | 40.5 | 51 | 38.6 | 104 | 39.5 |
| **Traffic jams on roads** | 30 | 28.0 | 36 | 29.5 | 66 | 28.8 |
| **Increased heavy traffic** | 55 | 51.4 | 62 | 50.8 | 117 | 51.1 |
| **Increased road accidents** | 22 | 20.6 | 24 | 19.7 | 46 | 20.1 |

**Table: A6. Impact of SEZ on Technology**

|  |  | Treatment Group | | Control Group | | Overall Sample | |
| --- | --- | --- | --- | --- | --- | --- | --- |
|  |  | N | % | N | % | N | % |
| **Transfer of technology** | **Yes** | 38 | 38 | 44 | 44 | 82 | 41 |
|  | **No** | 62 | 62 | 56 | 56 | 118 | 59 |
| **Transfer in different sectors** | **Agriculture sector** | 23 | 27.4 | 30 | 29.1 | 53 | 28.3 |
|  | **Commercial Sector** | 10 | 11.9 | 17 | 16.5 | 27 | 14.4 |
|  | **Industrial sector** | 26 | 31.0 | 29 | 28.1 | 55 | 29.4 |
|  | **Skilled labour** | 25 | 29.7 | 27 | 26.2 | 52 | 27.8 |

**Table: A7: Components, Eigenvalue and Data Variation**

| Component | Eigenvalue | Difference | Proportion of  variance explained | Cumulative variance explained |
| --- | --- | --- | --- | --- |
| **1** | 9.45 | 5.76 | 0.26 | 0.26 |
| **2** | 3.69 | 1.69 | 0.10 | 0.37 |
| **3** | 2.00 | 0.12 | 0.06 | 0.42 |
| **4** | 1.87 | 0.37 | 0.05 | 0.47 |
| **5** | 1.51 | 0.16 | 0.04 | 0.51 |
| **6** | 1.35 | 0.08 | 0.04 | 0.55 |
| **7** | 1.27 | 0.11 | 0.04 | 0.59 |
| **8** | 1.16 | 0.12 | 0.03 | 0.62 |
| **9** | 1.04 | 0.05 | 0.03 | 0.65 |
| **10** | 0.99 | 0.08 | 0.03 | 0.68 |
| **11** | 0.90 | 0.06 | 0.03 | 0.70 |
| **12** | 0.85 | 0.06 | 0.02 | 0.72 |
| **13** | 0.79 | 0.02 | 0.02 | 0.75 |
| **14** | 0.77 | 0.02 | 0.02 | 0.77 |
| **15** | 0.74 | 0.06 | 0.02 | 0.79 |
| **16** | 0.69 | 0.09 | 0.02 | 0.81 |
| **17** | 0.59 | 0.03 | 0.02 | 0.82 |
| **18** | 0.57 | 0.02 | 0.02 | 0.84 |
| **19** | 0.54 | 0.03 | 0.02 | 0.85 |
| **20** | 0.52 | 0.02 | 0.01 | 0.87 |
| **21** | 0.49 | 0.04 | 0.01 | 0.88 |
| **22** | 0.45 | 0.01 | 0.01 | 0.90 |
| **23** | 0.44 | 0.02 | 0.01 | 0.91 |
| **24** | 0.42 | 0.02 | 0.01 | 0.92 |
| **25** | 0.40 | 0.05 | 0.01 | 0.93 |
| **26** | 0.35 | 0.03 | 0.01 | 0.94 |
| **27** | 0.32 | 0.02 | 0.01 | 0.95 |
| **28** | 0.29 | 0.03 | 0.01 | 0.96 |
| **29** | 0.27 | 0.01 | 0.01 | 0.96 |
| **30** | 0.26 | 0.04 | 0.01 | 0.97 |
| **31** | 0.22 | 0.01 | 0.01 | 0.98 |
| **32** | 0.22 | 0.05 | 0.01 | 0.98 |
| **33** | 0.17 | 0.01 | 0.00 | 0.99 |
| **34** | 0.16 | 0.02 | 0.00 | 0.99 |
| **35** | 0.14 | 0.02 | 0.00 | 1.00 |
| **36** | 0.12 | . | 0.00 | 1.00 |

**Table: A8. Factor loading of PCA Model**

| Factor loadings | | | | | | | | | |
| --- | --- | --- | --- | --- | --- | --- | --- | --- | --- |
| **Variable** | **Factor 1** | **Factor 2** | **Factor 3** | **Factor 4** | **Factor 5** | **Factor 6** | **Factor 7** | **Factor 8** | **Factor 9** |
| **1** | 0.14 | -0.01 | -0.26 | -0.30 | -0.15 | 0.10 | 0.05 | -0.31 | 0.19 |
| **2** | 0.04 | 0.01 | -0.28 | -0.08 | 0.14 | 0.48 | 0.04 | 0.17 | 0.14 |
| **3** | 0.00 | 0.16 | 0.20 | 0.20 | -0.40 | 0.03 | -0.04 | 0.32 | 0.10 |
| **4** | -0.06 | 0.32 | 0.25 | -0.05 | -0.06 | -0.08 | 0.11 | -0.21 | 0.30 |
| **5** | 0.19 | -0.25 | -0.05 | 0.12 | 0.06 | 0.16 | -0.13 | 0.23 | -0.19 |
| **6** | 0.16 | 0.07 | 0.19 | -0.23 | 0.12 | 0.23 | 0.09 | 0.19 | 0.13 |
| **7** | 0.14 | -0.08 | 0.24 | 0.36 | -0.13 | -0.06 | 0.08 | -0.11 | 0.12 |
| **8** | 0.18 | -0.01 | -0.05 | -0.09 | -0.39 | 0.09 | -0.03 | 0.19 | -0.08 |
| **9** | 0.22 | -0.17 | -0.04 | 0.09 | -0.17 | 0.00 | -0.09 | 0.03 | 0.16 |
| **10** | 0.17 | -0.17 | -0.04 | 0.00 | 0.12 | -0.11 | -0.01 | 0.13 | 0.26 |
| **11** | 0.22 | 0.03 | -0.10 | -0.02 | -0.16 | 0.00 | -0.01 | 0.04 | 0.00 |
| **12** | 0.25 | 0.07 | 0.08 | -0.03 | -0.01 | 0.02 | 0.06 | -0.07 | -0.05 |
| **13** | 0.21 | 0.06 | 0.27 | -0.27 | 0.07 | 0.05 | -0.02 | 0.05 | -0.07 |
| **14** | 0.22 | -0.18 | -0.18 | -0.07 | 0.00 | -0.13 | -0.05 | 0.14 | 0.19 |
| **15** | 0.12 | 0.19 | -0.05 | -0.15 | -0.37 | -0.07 | 0.03 | 0.35 | -0.24 |
| **16** | 0.24 | 0.11 | 0.22 | -0.10 | 0.05 | -0.05 | -0.02 | -0.03 | -0.15 |
| **17** | 0.05 | -0.07 | -0.03 | 0.29 | 0.10 | -0.13 | 0.36 | 0.39 | 0.44 |
| **18** | 0.13 | 0.13 | 0.24 | -0.22 | 0.26 | 0.13 | -0.09 | 0.17 | 0.08 |
| **19** | 0.27 | -0.04 | -0.02 | -0.06 | 0.07 | -0.03 | 0.07 | -0.13 | -0.08 |
| **20** | 0.21 | -0.13 | 0.13 | 0.03 | 0.01 | -0.15 | 0.18 | -0.03 | -0.17 |
| **21** | 0.11 | -0.04 | -0.12 | 0.08 | -0.08 | 0.35 | 0.41 | -0.06 | -0.19 |
| **22** | 0.18 | -0.01 | -0.04 | -0.10 | 0.08 | -0.15 | -0.50 | 0.07 | 0.17 |
| **23** | 0.09 | -0.15 | 0.36 | -0.10 | 0.12 | -0.17 | 0.32 | 0.00 | 0.02 |
| **24** | 0.26 | -0.05 | 0.06 | 0.04 | -0.07 | -0.09 | -0.01 | -0.18 | -0.02 |
| **25** | 0.19 | -0.08 | -0.07 | 0.04 | -0.04 | -0.19 | -0.12 | -0.15 | 0.02 |
| **26** | 0.07 | 0.17 | 0.22 | 0.22 | 0.18 | 0.29 | -0.31 | 0.05 | 0.03 |
| **27** | 0.22 | -0.01 | -0.01 | -0.04 | -0.05 | 0.24 | 0.12 | -0.17 | 0.05 |
| **28** | 0.04 | -0.23 | -0.02 | 0.25 | 0.18 | -0.07 | -0.11 | 0.09 | -0.28 |
| **29** | 0.09 | 0.13 | -0.19 | 0.09 | 0.37 | 0.07 | 0.14 | 0.07 | -0.02 |
| **30** | 0.21 | -0.12 | 0.08 | 0.21 | 0.09 | 0.10 | 0.06 | -0.15 | -0.15 |
| **31** | -0.01 | 0.38 | -0.05 | 0.12 | 0.01 | -0.05 | 0.17 | 0.10 | -0.12 |
| **32** | 0.19 | 0.22 | -0.25 | 0.10 | 0.02 | -0.18 | 0.06 | -0.08 | 0.10 |
| **33** | 0.20 | 0.28 | -0.19 | 0.03 | 0.09 | -0.12 | 0.03 | -0.06 | 0.15 |
| **34** | 0.14 | 0.31 | 0.01 | 0.21 | 0.07 | -0.05 | -0.11 | 0.05 | -0.15 |
| **35** | 0.03 | 0.02 | 0.12 | 0.33 | -0.19 | 0.35 | -0.18 | -0.26 | 0.23 |
| **36** | 0.13 | 0.31 | -0.20 | 0.16 | 0.11 | -0.11 | -0.05 | -0.06 | -0.16 |
